# From amino acid analysis to improved gel properties: The role of dl-valine in Landaise goose myofibrillar protein

**DOI:** 10.1016/j.fochx.2024.101123

**Published:** 2024-01-09

**Authors:** Xinxin Yan, Hong Li, Xiujuan Wang, Zhonghai Hu, Jingjun Li, Haibo Zheng, Jie Wang, Zongyuan Zhen

**Affiliations:** a College of Food Engineering, Anhui Science and Technology University, Chuzhou 233100, China; b Huoqiu County Animal Health Supervision Institute, Lu’an 237400, China; c Lu'an Longxiang Gourmet Poultry Co., Ltd., Lu’an 237400, China; d WND Sci-Tech Development Service Center, Wuxi 214000, China; e The Institute of Functional Agriculture (Food) Science and Technology at Yangtze River Delta (iFAST), Chuzhou 239000, China; f Anhui Provincial Key Laboratory of Functional Agriculture and Functional Food, Chuzhou 233100, China

**Keywords:** Landaise goose, Myofibrillar protein, dl-valine, Gel properties, Low-field NMR

## Abstract

The impact of exogenous limiting amino acids on protein gel formation was investigated to enhance the gelation properties of Landaise goose myofibrillar protein (MP). Amino acid composition and gel properties were analyzed, and homologous protein modeling and molecular docking techniques were used to simulate binding sites. Valine was identified as the first limiting amino acid. The addition of 0.075 % dl-valine proved optimal to enhance the gel strength (59.5 g) and water retention (76.76 %) of MP gels. Hydrophobic interactions and disulfide bonds were found to be the main forces maintaining conformational stability of the MP-dl-valine gels. The propyl group of dl-valine can form hydrophobic interactions with protein, contributing to stable complexes. DL valine could also strengthen chemical bonds and secondary structure, convert free water to immobile water, and improve the microstructure of the gel. Therefore, valine can be utilized as a nutritional and gel enhancer in Landaise goose meat products.

## Highlights

• The limiting amino acid in Landaise goose muscle was found to be valine.
• The forces maintaining the conformation of goose protein gels were analyzed.
• The usefulness of DL valine as a gel fortifier was demonstrated.
• Molecular docking was used to build between DL valine and goose muscle proteins.
• The propyl group of DL valine forms hydrophobic interactions with amino acids.

## 1 Introduction

Goose meat is nutritionally-rich and high in essential amino acids and unsaturated fatty acids(Geldenhuys, Hoffman, & Muller, 2015). Landaise geese are reared worldwide primarily for their liver, but utilization of their muscle tissue is low. In addition, there has been minimal development of related gel products, despite the popularity of the category, because of the poor gelation quality of the muscle. Therefore, there is an urgent need for the development and improvement of products based on Landaise goose muscle tissues to improve the carcass utilization of this highly nutritious resource.

Myofibrillar protein (MP) plays a crucial role in muscle gel formation during thermal processing, is a major component of muscle and is well-known to form gels during thermal processing(Du et al., 2022a). Animal derived proteins can improve MP gel quality, resulting in improvements in the organoleptic properties and texture of meat products(Bai et al., 2023, Du et al., 2022b). Several amino acids, including l-lysine (l-Lys), l-arginine (l-Arg), and l-histidine (l-His), have been found to be effective in enhancing MP gels when exogenously applied by increasing the pH of the meat product and inhibiting protein aggregation(Shi et al., 2022). For goose protein, the limiting amino acids are primarily valine and methionine + cysteine, among others(Yan et al., 2023). dl-valine is the stereoisomer of l-valine and is widely used as a nutritional enhancer due to its low cost and safety. As one of the limiting amino acids in goose meat, dl-valine holds potential as a gel enhancer for goose meat and derived products.

This study aimed to determine whether exogenous limiting amino acids had an impact on MP gel derived from Landaise goose muscle. We first performed nutritional analysis to determine the limiting amino acids in the muscle proteins of Landaise geese. Next, we evaluated the protein solubility, chemical force, secondary structure, conformation and aggregated states, moisture state, and gel microstructure of the Landaise goose muscle MP gels after applying different proportions of exogenous limiting amino acids. In addition, homologous protein modeling and molecular docking analyses were conducted to study the interaction between dl-valine and Landaise goose myosin. This research will aid in the application of amino acids as gel enhancers in processed goose meat.

## 2 Materials and methods

### 2.1 Material preparation

We purchased 14 Landaise goose carcasses from Longxiang Gourmet King Poultry Co. Ltd. in Lu'an City, Anhui Province, China. The carcasses were randomly selected from the same batch of 80-day-old birds. The carcasses were frozen at −18 °C for subsequent analyses. From these, approximately 12 breast and leg meat samples were collected for testing. All experiments were completed within 48 h. To ensure experimental accuracy, each batch of experiments utilized muscle samples from the same geese to prepare protein gels.

### 2.2 Sample preparation

#### 2.2.1 Goose muscle processing

After thawing the fresh-frozen goose carcasses, excess tendon and fat were removed with a knife, and then breast and leg meat was excised completely. One portion of the meat was used for analysis of amino acid composition and other tests. The other portion was chopped into small uniform pieces and minced with a high-speed tissue mincer (Putian FK-A(JJ-2)). The resulting minced meat was used to extract MP.

#### 2.2.2 Extraction of myofibrillar protein

The procedure of Fan et al(Fan et al., 2023) was used to extract MP, with minor modifications. Briefly, 100 mL of stiffening solution (10 mmol/L sodium phosphate, 0.1 mol/L NaCl, 2 mmol/L MgCl_2_, 1 mmol/L EDTA, pH 7.0) was added to each 25 g sample of minced meat. The mixture was blended for 30 s at 10,000 rpm with a high-shear homogenizing emulsifier (Huyi HR500D). The homogenized mixture was then centrifuged at 4000 rpm with a high-velocity hypothermal centrifuge (Kecheng H4-20KR). After 5 min, the supernatant was decanted and 100 mL of stiffening solution was added to the precipitate; this process was repeated 3 times. Then, 100 mL of 0.1 mol/L NaCl solution was added to the resulting precipitate, and the mixture was homogenized at 10,000 rpm for 30 s; this process was repeated 3 times. The homogenized mixture was centrifuged for 5 min at 4000 rpm, then filtered through two layers of gauze. After repeating this operation 3 times, the filtrate was collected and calibrated by 0.1 mol/L HCl or NaCl to a pH of 6.25 (as determined with a Yoke p901 acidity meter). The pH-adjusted filtrate was centrifuged for 5 min at 4000 rpm, and the supernatant was discarded. The precipitate (myofibrillar protein) was collected and preserved between 0 °C and 4 °C for at most 48 h prior to use.

#### 2.2.3 Sample processing and preparation of thermally-induced gels

After dissolving the precipitate in 5–10 mL of 0.2 M phosphate buffer solution (PBS, 8.1 mM Na_2_HPO_4_, 1.9 mM NaH_2_PO_4_, 0.1 M NaCl, pH 7.0, 4◦C), the 50 mg/mL protein solution was adjusted by the biuret method (Zhu et al., 2023). The resulting mixture was divided into 6 treatment groups according to the amount of exogenous amino acids applied: 0 % (blank group), 0.025 %, 0.05 %, 0.075 %, 0.1 %, and 0.125 %. Three sets of parallel samples were produced per treatment group. Food-grade dl-valine (99.4 %) was acquired from Henan Wanbang Chemical Reagent Co. (Henan, China). The MP-exogenous amino acid mixtures were stirred slowly for 30 min until completely dissolved and left overnight (approx. 12 h) in the refrigerator (approx. 4 °C). The samples were then placed in 10 mL beakers, warmed from 20 °C to 80 °C using an electric thermostatic water bath, held at 80 °C for 30 min, promptly moved to an ice bath for 30 min, and then stored in a refrigerator (approx. 4 °C) for 24 h.

### 2.3 Testing methods

#### 2.3.1 Determination of amino acid composition

Approximately 9 breast and leg meat samples were used as parallel samples for amino acid composition analysis. Three parallel samples were collected from each carcass piece. The amino acid composition (g/100 g) was measured following the procedure of Boz et al.(Boz, Oz, Yamak, Sarica, & Cilavdaroglu, 2019) using a Hitachi automatic amino acid analyzer (L-8900). The amino acid compositions were scored (amino acid score, AAS) according to the evaluation model recommended by the FAO/WHO(Fao, 2013), as shown in Eq. (1).

(1) $$AAS\;=\;\frac{aa}{AA}\times 100\%$$

Where aa is the essential amino acid content (mg/g) and AA is the essential amino acid content of the reference protein (mg/g), as determined by FAO/WHO.

#### 2.3.2 Determination of gel chemical force

The method of Gómez-Guillén et al.(Gómez-Guillén, Borderías, & Montero, 1997) was employed to determine the chemical fore. Briefly, five samples of protein gel (2.0 g) were collected and individually mixed with either 0.05 mol/L NaCl (liquid A), 0.6 mol/L NaCl (liquid B), 0.6 mol/L NaCl + 1.5 mol/L urea (liquid C), 0.6 mol/L NaCl + 8.0 mol/L urea (liquid D), or 0.6 mol/L NaCl + 8.0 mol/L urea + 0.05 mol/L β-mercaptoethanol (liquid E). A 10 mL sample of each of these five solutions was homogenized for 60 min at 4 °C and then centrifuged for 15 min at 10,000 rpm. Next, the bicarbonate method was used to determine protein content of collected supernatant. The ionic bond content, hydrogen bond content, hydrophobic interactions, and disulfide bond content were calculated according to Eqs. (2), (3), (4), (5), respectively. The dissolved protein concentration was reported in grams per liter (g/L) as a result.

(2) Ionic bond content (g/L) = B-A

(3) Hydrogen bond content (g/L) = C-B

(4) Hydrophobic interactions (g/L) = D-C

(5) Disulfide bond content (g/L) = E-D

Where A represents the concentration of protein dissolved in liquid A (g/L), B represents the concentration of protein dissolved in liquid B (g/L), C represents the concentration of protein dissolved in liquid C (g/L), D represents the concentration of protein dissolved in liquid D (g/L), and E represents the concentration of protein dissolved in liquid E (g/L).

#### 2.3.3 Determination of protein secondary structure

We measured the protein secondary structure using Raman spectroscopy (HORIBA, XploRA PLUS), following the procedure of Feng et al.(Feng et al., 2023). Briefly, a slide was prepared with a sample weighing approximately 1.5 g and scanned under the following experimental conditions: laser wavelength = 532 nm, laser power = 100 mW, scanning range = 400–3600 cm^−1^, spectral resolution = 2.0 cm^−1^, number of scanned samples = 3, number of exposures = 60, data acquisition = 1 cm^−1^, speed = 120 cm^−1^/min. The results were normalized to phenylalanine (1003 cm^−1^) after determination. Analysis of the protein amide I band (1600–1700 cm^−1^) was conducted with Peakfit 4.12, with sequential baseline correction, deconvolution, and second-order derivative matching. Utilizing the method proposed by Alix et al.(Alix, Pedanou, & Berjot, 1988), subpeak area analysis was employed to calculate the relative content of each secondary structure category in the myogenic fibronectin.

#### 2.3.4 Determination of total sulfhydryl content and active sulfhydryl content

A slight modification of Chen et al.'s method(Chen et al., 2021) was utilized to measure the sulfhydryl content. Briefly, the mass concentration of the myofibrillar protein solution was adjusted to 5 mg mL^−1^. To each 1.5 mL sample of the adjusted solution was added 4.5 mL of buffer solution (0.2 mol/L Tris-HCL, pH 6.8; 8 mol/L urea; 2 % (w) SDS; 10 mmol/L EDTA). To each 4 mL sample of the mixed solution was added 0.25 mL of DTNB, and the mixture was water-bathed at 40 °C for 25 min. Subsequently, the absorbance was measured at 412 nm.

To determine the content of active sulfhydryl groups, 0.5 mL of adjusted solution was mixed with 4.5 mL of buffer solution (0.2 mol/L Tris-HCL, pH 6.8; 10 mmol/L EDTA). To this was added 0.25 mL of DTNB, and the mixture was reacted at 4 °C for 1 h. Subsequently, the absorbance was measured at 412 nm. The molar concentration of sulfhydryl groups was calculated according to the following formula.

(6) $$bSH=\frac{10^{6}\times A\times D}{13600\times C}$$

Where bSH is the molar concentration of thiol groups (mmol kg^−1^), A is the absorbance of the sample at 412 nm after removing the blank reagent, C is the protein mass concentration (mg mL^−1^), and D is the dilution factor. Each sample was repeated 3 times.

#### 2.3.5 Determination of surface hydrophobicity

The procedure of Chelh et al. (Chelh, Gatellier, & Sante-Lhoutellier, 2006) was employed to measure surface hydrophobicity, with slight modifications. Briefly, the MP-exogenous amino acid mixture was dissolved in 20 mmol/L phosphate buffer solution (pH 7.0) to prepare a protein solution with a mass concentration of 5 mg/mL. Then, 5 mL of MP solution was mixed evenly with 1 mL of bromophenol blue solution (1 mg/mL) and centrifuged at 6000 rpm for 15 min. We measured absorbance at 595 nm by diluting 10 times the supernatant with phosphate buffer, along with a blank control without MP. Surface hydrophobicity was calculated according to the quantity of bound bromophenol blue, as shown in formula (7).

(7) $$Amount\;of\;bound\;bromophenol\;blue/\mu g=\frac{\left(Ac-As\right)*200\mu g}{Ac}$$

Where c represents the control group, s represents the sample, and A is the absorbance value.

#### 2.3.6 Determination of average particle size and Zeta potential

Here, the procedure of Hu et al. (Hu et al., 2020) was utilized. Briefly, the MP was diluted to 0.1 mg/mL and 0.01 mg/mL, and a nanoparticle size potential analyzer was used to measure the particle size and Zeta potential of the respective diluted samples. Dynamic light scattering was utilized to measure the sample particle size at 25 °C, using a 4 mW helium/neon laser with a wavelength output of 633 nm and backscattering calibration at a detection angle of 173°. Zeta potential was measured using an electrophoretic mobility cell (DTS1070) at 25 °C.

#### 2.3.7 Determination of moisture state

It is widely accepted to study the moisture status of foods by Low-field NMR. Within the MP gel system, water exists in three states: (1) bound water, which is firmly restrained by MP molecules and exhibits a relaxation time (RT) between 1 and 10 ms (T2b); (2) immobile water, which is bound in gaps and holes in the MP gel network and exhibits a RT between 200 and 400 ms (T21); and (3) free water, which doesn't have a gel binding and is free to flow, with a RT between 1000 and 2000 ms (T22)(Wang et al., 2021).

The relaxation times of the gels were determined with a low-field NMR analyzer (NIUMAG NMI21-040H-I), following the procedure of Guo et al.(Guo et al., 2019). Briefly, the samples were cut into cubes (10 mm × 10 mm × 10 mm) of similar mass and packed into glass vials (mouth diameter <25 mm) for NMR analysis. The main parameters measured for T2 included: resonance frequency (MHz) = 20, TW (ms) = 4500, TE (ms) = 1.2, NS = 8, NECH = 8000. The Carr-Purcell-Mebbom-Gill (GPMC) sequence was run to determine spin-spin relaxation times (T2). Finally, we inverted the obtained plots to get the constituents of T2, viz. T2b, T22, and T23, and recorded the corresponding ratios, viz. PT2b, PT22, and PT23. Three measurements were conducted on each individual sample.

#### 2.3.8 Determination of MP gel texture

The intensity and elasticity of the gels were measured by a mass spectrometer (Stable MicroSystem TA-XT-PLUS) using the procedure of Zhu et al. (Zhu et al., 2023). The parameters used were: test probe = P/5, pre-test rate = l.0 mm/s, test rate = l.0 mm/s, puncture distance = 4 mm, sensing force = 5 g, data acquisition rate = 200.00 Hz. At least three parallel sets of samples were tested.

#### 2.3.9 Determination of the water retention of MP gel

The water holding capacity (WHC) of the gels was determined following the procedure of Shi et al.(Shi, Zheng, Liu, Gao, & Shao, 2021), with minor alterations. The prepared MP gels were freeze-centrifuged at 4000 rpm for 10 min and weighed. The water in the centrifuged samples was blotted with filter paper and then weighed using an electronic analytical balance (Mobil, MTQ500D). Three replicates of each treatment were measured and kept at 4 °C for testing. The water retention rate of the gel was evaluated using the following Eq. (8).

(8) WHC = (m_2_-m)/(m_1_-m) × 100 %

Where m is the centrifuge tube mass (g), m_1_ represents the pre-centrifuge mass of the gel and tube (g), and m_2_ is the post-centrifuge mass of the gel and tube (g).

#### 2.3.10 Determination of MP gel microstructure

Specimens were processed following the protocol of Hu et al.(Hu et al., 2023). Briefly, the prepared MP gels were cut into cuboids (0.5 cm × 0.4 cm × 0.3 cm), fixed with 2.5 % glutaraldehyde by volume for 2 d, and subsequently washed 3 times with phosphate buffer (pH 7.2) for 20 min. Next, the samples were eluted with gradients of 30 %, 50 %, 60 %, 70 %, 80 %, and 90 % ethanol by volume for 20 min, and then eluted 3 times with anhydrous ethanol for 30 min. Finally, the samples were dried, sprayed with gold, and evaluated at 2000× with a scanning electron microscope (ZEISS EVO-18) at 15.0 kV.

#### 2.3.11 Molecular docking analysis

The protein-ligand docking software Auto Vina was utilized to examine the binding energies and interactions among candidate small molecules and their targets. First, the three-dimensional structure of Landaise goose myosin was constructed through homology modeling of amino acid sequences acquired from NCBI and SWISS-MODEL. The 3D crystal structure was designated based on the model exhibiting the highest similarity and score. The protein crystal structure was acquired and preprocessed using the PDB database (https://www.rcsb.org/), and the plausibility of the 3D structural model was evaluated using MolProbity to obtain the Raschian conformation map(Kim et al., 2023). Next, the protein and ligand files were organized by conversion to the PDBQT format, exclusion of water molecules, and addition of polar hydrogen atoms. The grid box was positioned at the center to encompass the structural domains of each protein while allowing for unrestricted molecular movement. The docking pocket was set as a 30 Å×30 Å×30 Å square with a grid point distance of 0.05 nm. The molecular docking model was visualized with Autodock Vina 1.2.2 (https://autodock.scripps.edu/). Autodock version 1.5.6 was used in this study.

### 2.4 Data analysis

The recorded data were processed using Microsoft Excel, and the results were presented as the mean values accompanied by their respective standard deviations. Anova method was performed in SPSS 26.0 software to assess statistical significance, with a threshold of P < 0.05 indicating statistical significance. Origin 2021 was used to produce graphs and figures.

## 3 Results and discussion

### 3.1 Amino acid composition of Landaise goose muscle

Landaise goose muscles were found to contain a total of 17 amino acids (Table 1), with a total amino acid content of 18.82±0.91 g/100 g in the pectoral muscle and 19.95±1.35 g/100 g in the leg muscle. Glutamic acid stood out as the predominant amino acid, constituting 18.3 % of the overall amino acid composition. The glutamic acid contributed significantly to the umami taste of meat(Jiang et al., 2023). Essential amino acids accounted for 37.61 % and 38.37 % of Landaise goose pectoral and leg muscles, respectively, which is close to the ideal ratio determined by FAO/WHO (40 %)(Fao, 2013). Notably, the ratio for Landaise goose muscle is superior to that of other meat breeds, including the Polish Garbonosa and Chinese Jiangnan White Goose(Yan et al., 2023). The percentage of umami amino acids was similar to the average of 15 Chinese commercial goose breeds, indicating that the meat should have a good flavor and can be processed into palatable products.

**Table 1:** Amino acid composition of Landaise goose muscle tissues (g/100 g).

Amino acid	Breast muscle	Thigh muscle
Lys	1.74±0.10	1.85±0.12
Phe	0.79±0.05	0.82±0.06
Met	0.51±0.02	0.53±0.04
Ile	0.88±0.04	0.91±0.06
Leu	1.62±0.08	1.67±0.12
Thr	0.92±0.04	0.97±0.07
Val	0.89±0.04	0.91±0.06
Trp	0.19±0.01	0.21±0.01
Cys	0.05±0.01	0.05±0.00
Ala	1.21±0.06	1.26±0.10
Arg	1.29±0.06	1.41±0.09
Asp	1.81±0.09	1.91±0.13
Glu	3.41±0.19	3.62±0.28
Gly	0.86±0.07	1.01±0.10
His	0.71±0.04	0.70±0.02
Pro	0.45±0.02	0.56±0.06
Ser	0.79±0.04	0.85±0.06
Tyr	0.68±0.03	0.72±0.06
TAA	18.82±0.91	19.95±1.35
EAA/TAA	37.61 %	38.37 %
UAA/TAA	27.70 %	27.74 %

Table 1 demonstrates the composition (%) of each amino acid in Landaise goose thoracic and leg muscles. TAA stands for the total amino acid content. The essential amino acids (EAA) include Lys, Phe, Met, Ile, Leu, Thr, Val, Trp, and Cys. All other amino acids are considered non-essential amino acids (NEAA). The umami amino acids (UAA) include Asp and Glu.

The Landaise goose muscle amino acid composition and ratio, as well as the nutritional value, were highly satisfactory, with essential amino acid scores > 1 (Table 2). The greatest concentrations of amino acids in Landaise goose muscle tissues were threonine and lysine. The relative contents of threonine and lysine in the pectoral muscles were 4.88 % and 9.26 %, respectively, and in the hamstrings were 4.86 % and 9.26 %, respectively. Threonine is essential for nutrient metabolism regulation, macromolecular biosynthesis, and intestinal homeostasis. Among cereal grains like rice, lysine is recognized as the primary limiting essential amino acid, and lysine deficiency can result in malnutrition and weakened immunity(Yang et al., 2022). The higher content of lysine in Landaise geese can supplement lysine-deficient grain-based diets, and Landaise geese may be suitable for the development of dietary accompaniments to rice and other staple grains.

**Table 2:** Amino acid scores of Landaise goose muscle tissues.

Amino acid	FAO	Breast muscle	Thigh muscle
Thr	2.5	1.95	1.94
Val*	4	1.18	1.13
Ile	3	1.57	1.52
Leu	6.1	1.41	1.37
Lys	4.8	1.93	2.19
Try	0.66	1.53	1.59
Met + Cys	2.3	1.31	1.27
Phe + Tyr	4.1	1.9	1.89

Table 2 shows the amino acid scores (AAS) of Landaise goose meat compared to the FAO standard. Valine was the first limiting amino acid in Landaise goose meat.

The first limiting amino acid in Landaise goose muscle tissues is valine (Table 2), which is similar to the Polish Kielecka goose(Haraf et al., 2021) and the open-eyed goose(Yan et al., 2023). This is in contrast to other poultry, wherein the first limiting amino acid is often either methionine + cysteine or tryptophan. Valine is currently used as a food additive and nutritional fortifier in the form of dl-valine. dl-valine is used in nutritional supplements, pharmaceuticals, and flavorings, among other products, due largely to its affordability and convenience. However, to date, there have been no studies conducted on the impact of exogenous dl-valine on the gelation properties of meat. Therefore, we performed subsequent experiments to determine the effect of exogenously-applied dl-valine on the performance of Landaise goose MP gels.

### 3.2 Chemical force

**Fig. 1:**
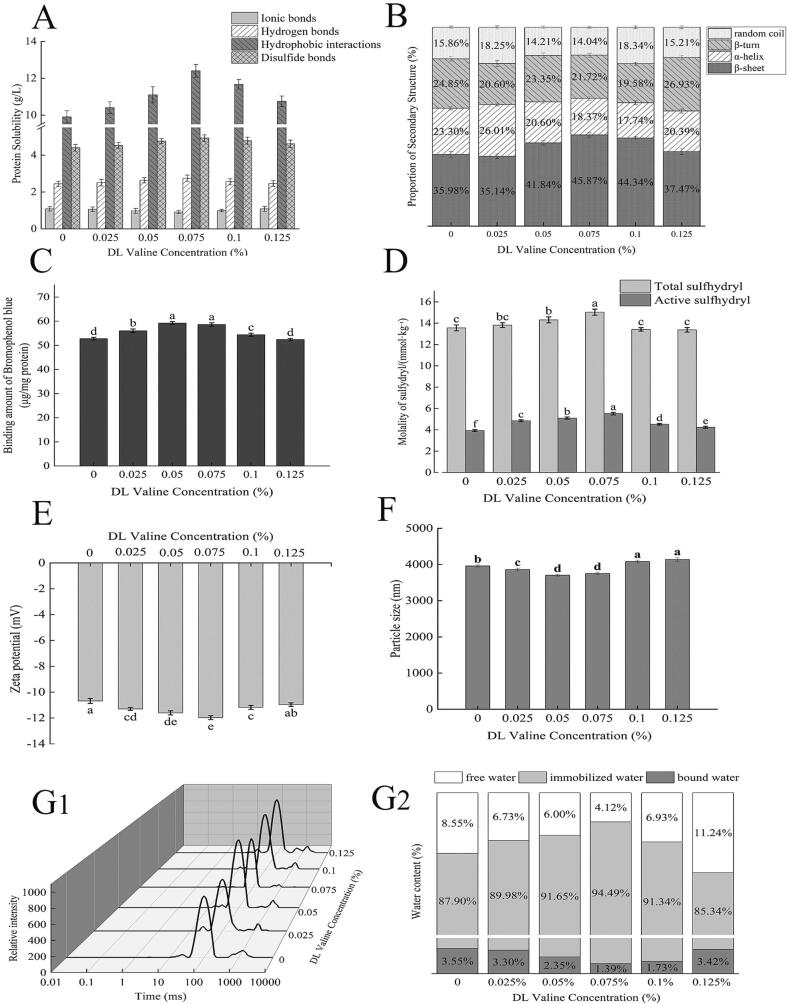
Influence of different DL valine concentrations on the chemical interactions (A), secondary structure (B), surface hydrophobicity (C), total sulfhydryl and active sulfhydryl (D), zeta potential (E), average particle size (F), T2 relaxation time (G_1_), and peak area ratio (G_2_) of Landaise goose MP gels. **A:** MP gels containing 0.075 % DL valine contained the highest proportion of hydrophobic interactions and disulfide bonds. Ionic and hydrogen bonds were not affected by the addition of DL valine. **B:** 0.075 % DL valine resulted in the highest β-sheet percentage and the lowest α-helix percentage observed in the gels, rendering these gels highly stable. At DL valine concentrations >0.075 %, the proportion of β-sheets decreased, rendering these gels less stable. With the addition of increasing concentrations of dl-valine, surface hydrophobicity **(C)**, the proportions of total and active sulfhydryl **(D)**, and the Zeta potential **(E)** tended to increase and then decrease. Conversely, the average particle size **(F)** tended to decrease and then increase. The dl-valine-MP gels exhibit a relatively stable state between 0.05 % and 0.075 % dl-valine.**G_1_ and G_2_:** Gels containing 0.075 % DL valine had the highest percentage of immobile water.

From weak to strong, the order of chemical forces in the Landaise goose MP gel was: ionic bonds < hydrogen bonds < disulfide bonds < hydrophobic interactions (Fig. 1A). These results indicate that hydrophobic interactions and disulfide bonds are the primary contributors to the preservation of conformational stability in MP gels. Neither the ionic nor hydrogen bonds changed significantly, while the concentration of dl-valine increased, leading to an initial rise in hydrophobic forces and a subsequent decline in the number of disulfide bonds. (P < 0.05) (Fig. 1). The importance of hydrophobic interactions in maintaining gel structure has been confirmed for other minced meat gels(Gao et al., 2021).

Chemical forces maintain the conformational stability of MP gels, and are closely related to strength, water retention, water distribution, and other gel properties (Zhang et al., 2023). Ionic and hydrogen bonds are weak intermolecular interactions and are easily destabilized by high temperatures, and therefore are not the primary chemical forces responsible for maintaining the stability of MP gels(Guan et al., 2023). Hydrogen bonding primarily maintains the secondary structures of proteins, and is responsible for transforming α-helices into β-sheets(Chen et al., 2020). Protein tertiary or quaternary structures are stabilized by ionic bonds that form between amino acid residues with opposing charges, attracting the Coulomb force(Mi et al., 2019). The addition of dl-valine promotes the unfolding of protein tertiary structures. Specifically, DL-valine’s propyl group promotes hydrophobic interactions and exposes hydrophobic groups such as tryptophan residues. Hydrophobic groups being exposed to polar environments facilitates the reaggregation of tail myogenic fibronectin to form stable complexes. Such protein rearrangement results in a homogeneous, porous mesh structure, which confers a favorable texture and the ability to retain water to the gel. At the same time, sulfhydryl groups become susceptible to oxidation as proteins unfold at the tertiary structure level, generating disulfide bonds that strengthen interprotein cross-linking and stabilize the gel(Zhou, Yang, Wang, Wei, & Li, 2019). As the dl-valine content increases, the contact between dl-valine and MP protein also increases, leading to reduced hydrophobic interactions within the gel due to exposure of hydrophobic groups. Nevertheless, excessive addition of dl-valine may cause the protein structure to be overly unfolded and disrupt crosslinks necessary for gel formation. In addition, the aggregation of excessive dl-valine and protein can occlude the sulfhydryl groups and prevent the generation of disulfide bonds, thus reducing the disulfide bond content of the gel.

### 3.3 Secondary structure

Increasing the content of dl-valine from 0 % to 0.075 % resulted in significantly improved MP gel properties (P < 0.05), as evidenced by a lower α-helix percentage and a higher β-sheet percentage (Fig. 1B). The maximum proportion of β-sheets (45.87 %) was observed at a dl-valine content of 0.075 %, indicating strengthened gelation and network formation. However, at a dl-valine content of 0.125 %, a large proportion of β-turns and random coils (42.14 %) was observed, which was unfavorable for the development of a well-organized gel mesh structure(Zhao, Zhou, & Zhang, 2019).

The β-sheet structure is large in surface area and weak in hydration ability, making it conducive to cross-linking between proteins. A growth in the amount of β-sheets indicates the presence of more hydrogen bonds, which helps to preserve protein conformational stability and establish a compact gel network structure(He et al., 2022). Based on the tertiary structure analysis, it can be deduced that the inclusion of dl-valine resulted in protein cross-linking and aggregation, leading to the development of a three-dimensional structure supported by hydrophobic groups. This process was accompanied by the uncoiling of α-helices, the unrolling of the structures, and the transformation into β-sheets. The conversion of α-helices to β-sheets promotes the release of hydrophobic residues, thereby increasing the strength of hydrophobic intermolecular interactions and enhancing the stability of the gel. Similarly, Cen et al.(Cen et al., 2022) demonstrated that gel structure and properties may be improved by exposing hydrophobic regions and sulfhydryl groups, as well as converting α-helices to β-sheets.

### 3.4 Conformational changes and aggregation behaviors

Surface hydrophobicity represents the number of hydrophobic amino acid residues distributed on the protein surface. Sulfhydryl groups are important for maintaining the stability of the spatial conformation of MP, and exposure of sulfhydryl groups can improve the gel structure(Gao et al., 2021). Active sulfhydryl groups exposed on the surface of protein molecules are necessary for many protein functions(Kang, Shang, Li, & Ma, 2022). Zeta potential is a direct index of protein surface charge and can be used to characterize the stability of protein solution systems(Wang et al., 2024). Particle size is a macroscopic manifestation of the size of protein aggregates and an important factor affecting the functional properties of MP(Niu et al., 2020).

Here, as we observed, the surface becomes more hydrophobic when the hydrophobic groups of MP are exposed, as DL valine is added (Fig. 1C). Meanwhile, MP and water molecules lose their interaction. As protein structure unfolds, the contents of total and active sulfhydryl groups increase (Fig. 1D). In addition, surface charge increases, leading to enhanced mutual repulsion between protein molecules and reduced assembling among protein molecules. As a result, the average protein particle size gradually decreases (Fig. 1E), leading to a more stable system with a higher Zeta potential(Li et al., 2021). However, as the amount of bound DL valine increases, the fully unfolded MP becomes over-exposed. In this case, the hydrophobic groups on the surface cause protein re-aggregation through covalent cross-linking and hydrophobic interactions, among other processes. This leads to the weakening of MP surface hydrophobicity (Fig. 1C), causing the sulfhydryl groups to again be occluded. Therefore, the contents of total and active thiol groups decreases (Fig. 1D) and the average protein particle size increases (Fig. 1E), which causes the stability of the protein solution decreased and leads to a weaker Zeta potential value (Fig. 1F).

### 3.5 Moisture distribution

With the addition of increasing concentrations of dl-valine, an initial rise in WHC and a subsequent decline was observed in gel (Fig. 1G1). Raising the dl-valine content from 0 % to 0.075 % yielded a significant growth in the immobile water content (P < 0.05) while concurrently decreasing the proportion of free water (Fig. 1G2), likely because protein or gel networks now bound the previously free water. At a dl-valine concentration of 0.075 %, the lowest relaxation times and relative content of free water were observed (P < 0.05). These findings suggest that much of the water is stably and tightly bound to the gel at a dl-valine concentration of 0.075 %. Increasing the DL valine concentration from 0.075 % to 0.125 % yielded a significant decrease (P < 0.05) in the immobile water content, along with a corresponding rise (P < 0.05) in the percentage of free water. Moderate amounts of dl-valine reduced the degree of free water in the gel and encouraged immobile water to bind to the protein. Bound water and immobile water ratios are significantly higher than the blank group (P < 0.05).

The measurement of relaxation time (T2) is a valuable approach for quantifying the presence and extent of free water, because shorter T2s are indicative of the presence of immobile water. Furthermore, shorter relaxation times are indicative of tighter binding between water molecules and the gel structure, leading to a denser myogenic fiber gel network. The exogenous dl-valine and MP interact primarily through hydrogen bonding, salt bridges, and hydrophobic interactions imposed during heat treatment. These interactions promote the binding and clustering of proteins, developing a tightly interconnected network structure, locking in a greater percentage of immobile water, and increasing gel WHC(He et al., 2022). During the beginning phase of gel network densification, a proportion of free water is converted to immobile water. The chemical force results indicate that the addition of excessive dl-valine caused a greater proportion of hydrophobic groups to be exposed to the polar environment. This process resulted in an excessively unfolded protein structure and weakened interactions between protein molecules, disrupting the cross-linking necessary for gel formation. These unfavorable conditions hinder the retention of water molecules within the gel, leading to a decline in both its texture and WHC. Similarly, Zhu et al. (Zhu et al., 2022) demonstrated a significant correlation (P < 0.05) between decreased WHC in thermally-induced gels and the transformation of immobile water into free water.

### 3.6 Texture and water retention

In general, the addition of dl-valine resulted in stronger gels, with the exception of the 0.125 % concentration (Table 3). With the addition of increasing concentrations of dl-valine, the gel strength and elasticity increased initially, but decreased thereafter (P < 0.05). Notably, the addition of 0.075 % dl-valine improved gel strength, elasticity, and water retention by 31.23 %, 22.59 %, and 15.1 %, respectively, compared with the blank group.

**Table 3:** Influence of different dl-valine concentrations on the texture and water retention properties of Landaise goose protein gels.

Concentration (%)	Strength (g)	Elasticity (nm)	WHC (%)
0	45.34±0.47d	2.09±0.02d	66.69±1.79d
0.025	47±1.41d	2.42±0.12bc	68.71±1.61cd
0.05	52.3±1.25b	2.52±0.24ab	72±1.14b
0.075	59.5±1.12a	2.7±0.15a	76.76±1.25a
0.1	49.5±0.5c	2.36±0.11bc	70.38±1.42bc
0.125	41.34±0.47e	2.29±0.13bc	62.71±0.94e

Table 3 shows the effects of different concentrations (0, 0.025 %, 0.05 %, 0.075 %, 0.1 %, and 0.125 %) of dl-valine on the strength, elasticity, and water holding capacity (WHC) of Landaise goose MP gels. The most optimal results were obtained after the addition of 0.075 % dl-valine.

Strength and elasticity are key characteristics of MP gels, and provide valuable insights into the textural characteristics and quality traits of meat products. The WHC serves as an indicator of the moisture retention ability of a meat product, reflecting the protein structure and the extent of protein-water interaction within the gel network. Modifications in the secondary structure of MP gels result in improvements in their strength and elasticity. These enhancements can be attributed to increased hydrophobic interactions and stronger ionic bonding, which induce the overall textural characteristics of the gels. The elasticity and strength increased and then decreased to different degrees with the introduction of varying levels of dl-valine. Combined with the chemical bonding results, it appears that the active hydroxyl group of dl-valine encouraged the MP tertiary structure to unfold. The active hydroxyl group of dl-valine interacts with the hydrophobic groups of the protein, leading to a hydrophobic interaction that contributes to the stabilization of the gel structure. Our secondary structure analysis confirmed that the addition of dl-valine modified the side chains of the MP, unfolded the protein structures, promoted the uncoiling of α-helices and turned them into β-sheets, and formed a denser and more stable gel with improved texture and water retention. However, the excessive addition of dl-valine exceeded the binding limit, which was adverse to forming the network structure and led to worsened gel properties. A similar study on mutton-based MP gels reported consistent results regarding gel texture and water retention(Zhu et al., 2023).

### 3.7 Microstructure

**Fig. 2:**
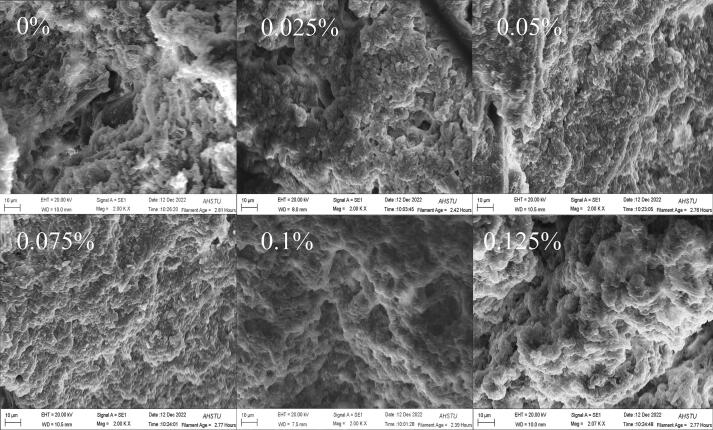
Influence of different DL valine concentrations on the microstructure of Landaise goose MP gels. Gels containing 0.075 % DL valine exhibited superior microstructure, with a uniform surface, small cavities, and a relatively dense structure. Gels containing 0.1 % and 0.125 % DL valine exhibited larger, unevenly distributed surface pores, with an inferior internal structure.

Different concentrations of dl-valine showed significant effects on the microstructure of goose MP protein gels (Fig. 2). In general, the more homogeneous, dense, and orderly the network structure, the more regular and flatter the microstructure and the more favorable the texture and WHC of the gel. Blank gels (0 % dl-valine) exhibited a porous, irregular microstructure, with a rough and uneven surface. The addition of dl-valine led to a drop in pore size and a denser gel structure. At a dl-valine concentration of 0.075 %, the gel exhibited a dense lamellar microstructure with a smooth and uniform surface and relatively few cavities, enhancing the WHC. These results indicate that dl-valine added at a suitable concentration can promote the development of MP gel networks. However, the excessive introduction of dl-valine (0.1 %, 0.125 %) resulted in larger pores, a rougher surface, and an overall looser gel structure. Our microstructure results are generally in agreement with our texture and water retention results.

### 3.8 Molecular docking

**Fig. 3:**
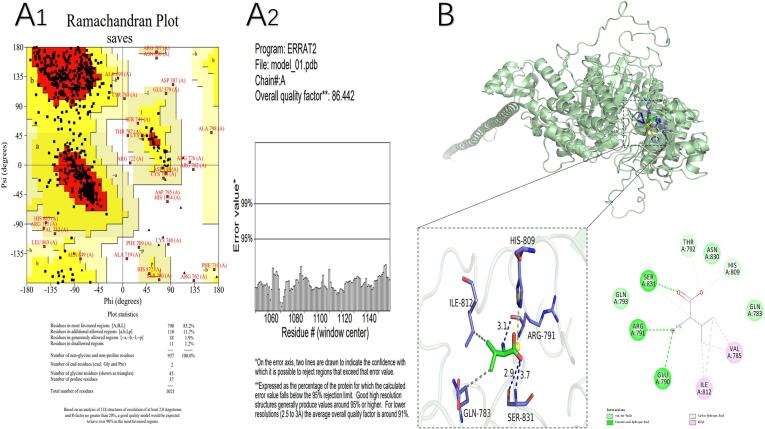
Evaluation of the Landaise goose myosin model (A_1_ and A_2_) and molecular docking results of dl-valine and Landaise goose myosin (B). A_1_: pull-down concept map. A2: protein model validation. Fig. 3A1 shows that nearly 100 % of amino acid residues are in the reasonable region. Fig. 3A2 shows that the protein model (model score: 86.442) meets ERRAT validation and the requirements of the evaluation >80 % procedure, proving the feasibility of the model. Fig. 3B shows the macroscopic view of molecular docking binding sites. Below the pictures are a bar diagram of molecular docking and a line diagram of molecular docking-specific binding sites. DL valine interacts with myosin mainly through hydrogen bonding, salt bridges, and hydrophobic interactions. The binding energy analysis indicates that dl-valine can bind to protein spontaneously.

The model obtained by Robetta homology modeling shows the calculated Raman pattern (Fig. 3A1). From this Raman conformation, we can see that there are only 1.2 % of amino acid residues located in disallowed regions, with nearly 100 % located within the reasonable region (85.2 % in the optimal region, 11.7 % in the acceptable region, and 1.9 % in the general permissive region). Therefore, according to the criteria that the sum of the reasonable region and the allowed region should exceed 90 %, the model was deemed successful and was used for further docking analysis. The protein model with the model score of 86.442 was found to meet ERRAT validation, as well as the requirements of the evaluation > 80 % procedure, proving the feasibility of the model (Fig. 3A2). The combined Ramachandran plot and ERRAT score highlight the reliability of the constructed protein structure model for use as a template in subsequent studies.

We used VINA 1.1.2 to study the docking of dl-valine and MP, which uses a semi-empirical free ability field to present the connecting energy of receptor and ligand. The binding energy was calculated to be −4.8 kcal/mol, which is less than the −4 kcal/mol threshold, indicating that dl-valine can spontaneously bind to MP (Fig. 3B). Subsequently, PYMOL was used to visualize whether dl-valine was stably bound to the MP cavity as well as the nature of its interactions with neighboring amino acids. dl-valine primarily engages in protein interactions through hydrogen bonds, salt bridges, and hydrophobic interactions (Fig. 3B). Specifically, dl-valine is able to form stable hydrogen bonds with ARG791 and SER831. These hydrogen bonds strengthen their non-covalent interactions and solidify their binding. Furthermore, the propyl group of dl-valine can form hydrophobic interactions with GLN83 and ILE812, which promotes the formation of stable compounds and induces the rearrangement of hydrogen bonds among the secondary structural components of the polypeptide chain. These processes result in a reduced α-helix composition, as well as a simultaneous elevated β-sheet structure, thus producing a more stable conformation. Overall, consistency was observed between the docking results and secondary and tertiary structure results. The positively charged HIS809 forms salt bridges with the carboxyl group of dl-valine, and this is the primary force inducing dl-valine to bind to the active site. The very small value associated with the electrostatic force indicates that it is not one of the main forces, although it does contribute to the maintenance of conformational stability.

In recent years, molecular docking has been widely used to obtain new drug candidates in a short time and at a low cost. Molecular docking experiments are useful to understand how ligands interact with receptors in various orientations, conformations, and positions, and can be used to produce comprehensive models of receptor-ligand interactions. The primary goal of most molecular docking experiments is to determine how a specific ligand attaches to a receptor protein with a known three-dimensional structure(Shao, Wang, Hu, Xu, & Wang, 2023). Future targeted experiments should be conducted to evaluate the interactions between dl-valine and arginine, tryptophan, glutamine, isoleucine, and histidine, as well as how these interactions alter protein conformation and gel structure.

### 3.9 Mechanism of dl-valine affecting MP gelation

As a non-polar amino acid, dl-valine interacts with MP mainly through hydrophobic interactions and disulfide bonds, unlike basic amino acids, which mainly work by adjusting the pH of the protein system(Ning et al., 2019). The propyl portion of dl-valine interacts hydrophobically with amino acids in proteins (Fig. 3), establishing stable complexes and affecting the conformation and aggregation of MP. MP is unfolded while its hydrophobic groups are exposed, which increases surface hydrophobicity and thiol groups contents (total and active), and reduces the average protein particle size, resulting in an increased Zeta potential values (Fig. 1C – F). Meanwhile, the unfolding of MP exposes active sulfhydryl groups, contributing to the generation of more disulfide bonds through oxidation as heating (Fig. 1A). Disulfide bonds combined with hydrophobic interactions result in changes to the secondary structure. The α-helices are converted into β-sheets (Fig. 1B), which promotes the generation of a more concentrated microscopic network structure (Fig. 2) and improves water distribution within the gel (Fig. 1G). These changes affect gel properties such as strength, elasticity, and water retention (Table 3).

## 4 Conclusion

We analyzed the amino acid content of Landaise goose muscle tissues and investigated the impacts of exogenous amino acid application on the MP conformation and aggregated states (sulfhydryl content, surface hydrophobicity, Zeta potential values, and particle size), gels properties (texture, water retention, chemotactic force, water status, microstructure, protein secondary structure), and intermolecular interaction of Landaise goose meat-based MP gels. The results showed that valine was the limiting amino acid, which can significantly affect the quality of Landaise goose meat. The gelation properties of the MP gels were strengthened by the addition of dl-valine in appropriate amounts, with a dl-valine content of 0.75 % proving optimal. MP-dl-valine gels maintain their conformational stability primarily via hydrophobic interactions and disulfide bonds. Molecular docking calculation indicated that dl-valine and myosin interacted principally through hydrogen bonds, salt bridges, and hydrophobic interactions. This study demonstrates the potential of dl-valine to enhance the quality of goose meat through nutritional fortification and to improve MP gelation properties.

## CRediT authorship contribution statement

**Xinxin Yan:** Conceptualization, Data curation, Formal analysis, Investigation, Methodology, Software, Writing – original draft, Writing – review & editing. **Hong Li:** Data curation, Formal analysis, Investigation. **Xiujuan Wang:** Investigation, Methodology. **Zhonghai Hu:** Funding acquisition, Project administration, Resources. **Jingjun Li:** Funding acquisition, Project administration. **Haibo Zheng:** Methodology, Validation. **Jie Wang:** Investigation, Validation. **Zongyuan Zhen:** Conceptualization, Data curation, Formal analysis, Funding acquisition, Investigation, Methodology, Project administration, Resources, Supervision, Writing – original draft, Writing – review & editing.

## Declaration of competing interest

The authors declare that they have no known competing financial interests or personal relationships that could have appeared to influence the work reported in this paper.

## Data Availability

Data will be made available on request.
